# PREDOMOS study, impact of a social intervention program for socially isolated elderly cancer patients: study protocol for a randomized controlled trial

**DOI:** 10.1186/s13063-017-1894-7

**Published:** 2017-04-12

**Authors:** Elodie Crétel-Durand, Emilie Nouguerède, Hervé Le Caer, Frédérique Rousseau, Frédérique Retornaz, Olivier Guillem, Anne-Laure Couderc, Laurent Greillier, Emmanuelle Norguet, Maud Cécile, Rabia Boulahssass, Francoise Le Caer, Sandrine Tournier, Chantal Butaud, Pierre Guillet, Sophie Nahon, Laure Poudens, Sylvie Kirscher, Sandrine Loubière, Nadine Diaz, Jean Dhorne, Pascal Auquier, Karine Baumstarck

**Affiliations:** 1 Unit of Transversal Onco-Geriatry (UTOG), Service de Médecine Interne et Gériatrie Thérapeutique, 264 Rue Saint Pierre, 13385 Marseille cedex 05, France; 2 Unit of Coordination in Onco-Geriatry (UCOG), PACA-west, Marseille, France; 3 CRO2 UMR_S 911, INSERM, Aix-Marseille Université, 27 Boulevard Jean Moulin, 13385 Marseille cedex 05, France; 4 Service de Pneumologie, CH Saint Brieuc - Hôpital Ives Le Foll, 10 Rue Marcel Proust, 22000 Saint Brieuc, France; 5 Service d’Oncologie Médicale, Institut Paoli Calmettes, 232 Boulevard de Sainte Marguerite Dromel, 13009 Marseille, France; 6 Centre de Gérontologie Départemental, 176 Avenue de Montolivet, 13012 Marseille, France; 7 Service d’Onco-Gériatrie, CH Intercommunal des Alpes du Sud Site de Gap (CHICAS), 1 Place Auguste Muret, 05000 Gap, France; 8 Service de Médecine Interne et Gériatrie Thérapeutique, Hôpital Sainte Marguerite, Assistance Publique des Hôpitaux de Marseille (AP-HM), 270 Boulevard de Sainte Marguerite Dromel, 13274 Marseille cedex 09, France; 9 Oncologie Multidisciplinaire et Innovation Thérapeutique, CHU NORD, Assistance Publique des Hôpitaux de Marseille (AP-HM), Chemin des Bourrely, 13915 Marseille cedex 20, France; 10 Service d’Oncologie Digestive, CHU Timone, Assistance Publique des Hôpitaux de Marseille (AP-HM), 264 Rue Saint Pierre, 13385 Marseille cedex 05, France; 11 Service de Gérontologie, Hôpital de Cimiez, 4 Avenue Reine Victoria, CS 91179, 06003 Nice, France; 12 Pôle de Gériatrie, CH Saint Brieuc, Hôpital Yves Le Foll, 10 Rue Marcel Proust, 22000 Saint Brieuc, France; 13 Service de Gériatrie, Hôpital Saint Joseph, 26 Boulevard Louvain, 13285 Marseille cedex 08, France; 14 Unité Mobile de Gériatrie, Hôpital Saint Musse, CH Intercommunal Toulon-La Seyne sur Mer (CHITS), 54 Rue Henri Claire Deville, 83000 Toulon, France; 15 Service d’Hémato-Oncologie, CH du Pays d’Aix, Avenue les Tamaris, 13616 Aix-en-Provence, France; 16 Service d’Oncologie Médicale, Institut Sainte Catherine (ISC), 250 Chemin de Baigne Pieds, 84918 Avignon cedex 09, France; 17 EA3279, Self-perceived Health Assessment Research Unit, Aix-Marseille University, 27 Boulevard Jean Moulin, 13385 Marseille cedex 05, France; 18 Service Social, Hôpital Sainte Marguerite, Assistance Publique des Hôpitaux de Marseille (AP-HM), 270 Boulevard de Sainte Marguerite Dromel, 13274 Marseille cedex 09, France; 19 Direction de la Recherche Clinique et de l’Innovation (DRCI), Assistance Publique des Hôpitaux de Marseille (AP-HM), 80 Rue Brochier, 13354 Marseille Cedex 05, France

**Keywords:** Social intervention program, Techniques for domotic and remote assistance, Elderly cancer, Oncogeriatrics, Randomized controlled trial

## Abstract

**Background:**

Cancer incidence and social isolation increase along with advanced age, and social isolation potentiates the relative risk of death by cancer. Once spotted, social isolation can be averted with the intervention of a multidisciplinary team. Techniques of automation and remote assistance have already demonstrated their positive impact on falls prevention and quality of life (QoL), though little is known about their impact on socially isolated elderly patients supported for cancer.

The primary objective of the PREDOMOS study is to evaluate the impact of establishing a Program of Social intervention associated with techniques of Domotic and Remote assistance (PS-DR) on the improvement of QoL of elderly isolated patients, treated for locally advanced or metastatic cancer. The secondary objectives include treatment failure, tolerance, survival, and autonomy.

**Methods/design:**

This trial is a multicenter, prospective, randomized, placebo-controlled, open-label, two-parallel group study. The setting is 10 French oncogeriatric centers. Inclusion criteria are patients aged at least 70 years with a social isolation risk and a histological diagnosis of cancer, locally advanced or metastatic disease. The groups are (1) the control group, receiving usual care; (2) the experimental group, receiving usual care associating with monthly social assistance, domotic, and remote assistance. Participants are randomized in a 1:1 allocation ratio. Evaluation times involve inclusion (randomization) and follow-up (12 months). The primary endpoint is QoL at 3 months (via European Organization for Research and Treatment of Cancer (EORTC) QLQ C30); secondary endpoints are social isolation, time to treatment failure, toxicity, dose response-intensity, survival, autonomy, and QoL at 6 months. For the sample size, 320 individuals are required to obtain 90% power to detect a 10-point difference (standard deviation 25) in QoL score between the two groups (20% loss to follow-up patients expected).

**Discussion:**

The randomized controlled design is the most appropriate design to demonstrate the efficacy of a new experimental strategy (Evidence-Based Medicine Working Group classification). National and international recommendations could be updated based on the findings of this study.

**Trial registration:**

ClinicalTrials.gov, NCT02829762. Registered on 29 June 2016.

**Electronic supplementary material:**

The online version of this article (doi:10.1186/s13063-017-1894-7) contains supplementary material, which is available to authorized users.

## Background

In France, social isolation and prevalence of cancer increase with population aging: it is estimated that in 2050, one out of two cancers will be diagnosed in patients more than 75 years old. At the same time, the number of isolated elderly increased one and a half fold between 2010 and 2013, and among persons living alone, one out of two is more than 60 years old [1]. Moreover, acute isolation of elderly patients coincides with the beginning of old age dependency, when support is most needed. Socially precarious elderly persons have an increased risk of dying of cancer [2–5], along with its increasing incidence in persons more than 65 years old [6]. Thus, elderly isolated patients represent a particularly sensitive population.

Comprehensive Geriatric Assessment (CGA) allows evaluation of geriatric frailties such as functional reserves and cancer-induced complications; it leads to the diagnosis of unsuspected conditions in 50% of cases [7]. To detect geriatric frailties, the International Society of Geriatric Oncology (SIOG) recommends to screen all patients older than 70 years with a specific scale (G8 scale) [8], and patients whose score is below a prefixed cut should be addressed to a geriatrician and benefit from a full CGA [8–12]. Among CGA domains, evaluation of social interactions holds a major role and can be evaluated by the modified Medical Outcomes Study Social Support Survey (m-MOS-SS) scale [13].

Once detected, social isolation can be avoided with the use of a multidisciplinary approach leading to adequate preventive actions [1]. Loss of autonomy, which is a major challenge for the elderly, is favored by social isolation, increasing their vulnerability. The preventive role of a social network can be partially substituted: automation and remote assistance techniques have already demonstrated their impact on falls prevention, autonomy loss, feelings of social isolation, and quality of life (QoL) [14, 15]. However, little is known about the benefit of establishing a social intervention associated with techniques of domotic and remote assistance for elderly patients, isolated or at risk of isolation, treated for locally advanced or metastatic cancer.

These observations prompted us to establish a multicenter, prospective, two-group, open-label, randomized study with the primary objective of assessing the impact of a Program of Social intervention of Domotic and Remote assistance (PS-DR) on the QoL of isolated elderly patients with cancer. The secondary objectives are to assess this new strategy in terms of treatment failure, tolerance and compliance, survival, physical status, autonomy, and occurrence of hospitalization.

## Methods/design

### Design

**Fig. 1:**
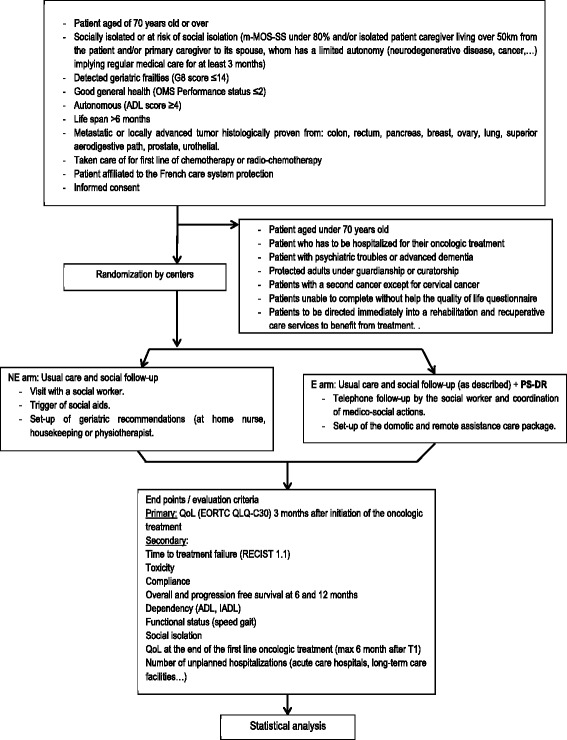
Study flow chart

This multicenter, prospective, randomized (1:1 ratio), open-label, two-parallel group study is performed to assess the interest of a Program of Social intervention including Domotic and Remote assistance (PS-DR, experimental group) compared to usual care. Figure 1 shows the study flow chart. The study protocol was designed using the recommendations of the Standard Protocol Items: Recommendations for Interventional Trials (SPIRIT) guidelines. (See Additional file 1 for the SPIRIT checklist 2013 statement).

### Partners

The sponsor of the study is the Assistance Publique des Hôpitaux de Marseille (AP-HM, France), which is in charge of the data monitoring. As no adverse events are expected from the intervention, there will be no need for a data monitoring committee. The recruitment will be performed in 10 French geriatric departments, belonging to the Unit of Coordination in Onco-Geriatry from the west Provence-Alpes-Côte d’Azur region (UCOG PACA-West) for 8 centers, the UCOG Bretagne for 1 center and to the UCOG PACA-East for 1 center. Methodological support will be provided by the Clinical Research Unit (Unité Aide Méthodologique à la Recherche Clinique, AP-HM, France), the Clinical Investigation Unit (Centre d’Investigation Clinique, AP-HM, France), and the Self-perceived Health Assessment Research Unit (Aix-Marseille University, Marseille, France). This study is supported by a grant from the French Ministry of Health. All the details are provided in Table 1.

**Table 1:** French partners

Physicians	Role in the study	Center/department
Dr Elodie CRÉTEL-DURAND	Coordinating investigator.	Marseilles’ University Hospital (AP-HM), Onco-Geriatic Transversal Unit (UTOG), Intern medicine and therapeutic geriatry Department, UCOG PACA-West, Marseille
Dr Laurent GREILLER	Principal investigator.	Marseilles’ University Hospital (AP-HM), Multidisciplinary Oncology and Therapeutic Innovations Department, Marseille
Pr Patrick VILLANI	Associated investigator.	Marseilles’ University Hospital (AP-HM), Intern medicine and therapeutic geriatry Department, Marseille
Dr Anne-Laure COUDERC	Associated investigator.	Marseilles’ University Hospital (AP-HM), Onco-Geriatic Transversal Unit, Intern medicine and therapeutic geriatry Department, UCOG PACA-West, Marseille
Pr Fabrice BARLESI	Associated investigator.	Marseilles’ University Hospital (AP-HM), Multidisciplinary Oncology and Therapeutic Innovations Department, Marseille
Dr Marie-Eve GARCIA	Associated investigator.	Marseilles’ University Hospital (AP-HM), Multidisciplinary Oncology and Therapeutic Innovations Department, Marseille
Dr Clothilde DE LEQUESNE	Associated investigator.	Marseilles’ University Hospital (AP-HM), Multidisciplinary Oncology and Therapeutic Innovations Department, Marseille
Dr Marjorie BACHIUCHKA	Associated investigator.	Marseilles’ University Hospital (AP-HM), Multidisciplinary Oncology and Therapeutic Innovations Department, Marseille
Pr Laetitia DAHAN	Associated investigator.	Marseilles’ University Hospital (AP-HM), Digestive Oncology Department, Marseille
Pr Jean-François SEITZ	Associated investigator.	Marseilles’ University Hospital (AP-HM), Digestive Oncology Department, Marseille
Dr Emmanuelle NORGUET-MONEREAU	Associated investigator.	Marseilles’ University Hospital (AP-HM), Digestive Oncology Department, Marseille
Dr Maud CECILE	Principal Investigator.	Paoli Calmette Institute, Department of Medical Oncology, UCOG PACA-West, Marseille
Dr Frédérique ROUSSEAU-EXTRA	Associated Investigator.	Paoli Calmette Institute, Department of Medical Oncology, UCOG PACA-West, Marseille
Dr Cécile BRATICEVIC	Associated Investigator.	Paoli Calmette Institute, Department of Medical Oncology, UCOG PACA-West, Marseille
Dr Louis TASSY	Associated Investigator.	Paoli Calmette Institute, Department of Medical Oncology, Marseille
Dr Hervé LE CAER	Principal Investigator.	Yves Le Foll hospital center, Saint Brieuc
Dr Françoise LE CAER	Associated Investigator.	Yves Le Foll hospital center, Saint Brieuc
Dr Gwenaëlle LE GRAFF	Associated Investigator.	Yves Le Foll hospital center, Saint Brieuc
Dr Hélène LE HÔ	Associated Investigator.	Yves Le Foll hospital center, Saint Brieuc
Dr Christine PIROT	Associated Investigator.	Yves Le Foll hospital center, Saint Brieuc
Dr AnnAïck PESTEL	Associated Investigator.	Yves Le Foll hospital center, Saint Brieuc
Dr Corine HAUCHART	Associated Investigator.	Yves Le Foll hospital center, Saint Brieuc
Dr Jean-Bernard DELOBEL	Associated Investigator.	Yves Le Foll hospital center, Saint Brieuc
Dr Corine ALLEAUME	Associated Investigator.	Yves Le Foll hospital center, Saint Brieuc
Dr Frédérique RETORNAZ	Principal Investigator.	Centre Gérontologique Dépatemental, UCOG PACA-West, Marseille
Dr Margaux VIEILLARD	Associated Investigator.	Centre Gérontologique Dépatemental, Marseille
Dr Chantal BUTAUD	Principal Investigator.	CHITS Sainte Musse Hospital, Mobile Geriatric Care Unit, UCOG PACA-West, Toulon
Dr Pierre GUILLET	Associated Investigator.	CHITS Sainte Musse Hospital, Oncology-Hematology Department, Toulon
Dr Sylvie KIRSCHER	Principal Investigator.	Saint Catherine Institute, Medical Oncology Department, UCOG PACA-West, Avignon
Dr Philippe DEBOURDEAU	Associated Investigator.	Saint Catherine Institute, Avignon
Dr Rania BOUSTANY	Associated Investigator.	Saint Catherine Institute, Oncology-Radiotherapy Department, Avignon
Dr Olivier GUILLEM	Principal Investigator.	South Alpes Intercomunal Hospital Center (CHICAS), Oncogeriatry Department, UCOG PACA-West, Gap
Dr Corrine GAILLARD	Associated Investigator.	South Alpes Intercomunal Hospital Center (CHICAS), Medical geriatry Department, Gap
Dr Sophie NAHON	Principal Investigator.	Pays d’Aix Hospital center, Hemato-Oncology Department, UCOG PACA-West, Aix-en-Provence
Dr Laure POUDENS	Associated Investigator.	Pays d’Aix Hospital center, Mobile Geriatric care Team, UCOG PACA-West, Aix-en-Provence
Dr Sandrine TOURNIER	Principal Investigator.	Saint Jospeh Hospital, Geriatry Department, UCOG PACA-West, Marseilles
Dr Eve YOUSSOF	Associated Investigator.	Saint Joseph Hospital, Oncology Day Hospital, Marseille
Dr Christelle BASTHISTE-PELE	Associated Investigator.	Saint Joseph Hospital, Oncology Day Hospital, Marseille
Dr Jean-Baptiste PAOLI	Associated Investigator.	Saint Joseph Hospital, Oncology Day Hospital, Marseille
Dr Hervé PERRIER	Associated Investigator.	Saint Joseph Hospital, Oncology Day Hospital, Marseille
Dr Cyril FOA	Associated Investigator.	Saint Joseph Hospital, Oncology Day Hospital, Marseille
Dr Rabia BOULAHSSASS	Principal Investigator.	Nice University Hospital, UCOG PACA-East, Nice
Dr Marine SANCHEZ	Associated Investigator.	Nice University Hospital, Nice
Multidisciplinary team		
Pr Pascal AUQUIER	Methodology	Public health, public academic teaching hospital, Marseille
Dr Karine BAUMSTARCK	Methodology	Clinical research unit, public academic teaching hospital, Marseille
Dr Emilie NOUGUEREDE	Study Coordinator	Marseilles’ Comprehensive Cancer center (SIRIC), Marseilles’ University Hospital (AP-HM)
Jean DHORNE	Project Manager	Clinical research and system innovation (DRCI), Marseilles’ University Hospital (AP-HM)

### Steering committee

Study conception and case report forms are conceived in collaboration with a steering committee composed of oncologists, social workers, and geriatricians. Clinicians comprise the major part of the UCOG PACA-West steering committee, which is in charge of the harmonization and diffusion of oncogeriatric care standards and guidelines in accordance with SIOG and French National Cancer Institute (INCa) guidelines [8, 9]. The steering committee is composed of oncologists and geriatricians from nine different hospitals.

### Participants

The details of the inclusion and exclusion criteria are provided in Table 2. The main inclusion criteria are patients more than 70 years old with a histological diagnosis of locally advanced or metastatic cancer (tumors of the lung, colon-rectum, pancreas, prostate, urothelium, ovary, breast, or upper aerodigestive tract) in first therapeutic line; defined as requiring a CGA based on the SIOG recommendations (G8 score ≤14); in good health conditions according to the World Health Organization (WHO) performance status (WHO score ≤2); who are autonomous (Activities of Daily Living (ADL) Scale score ≥4; Katz scale); and at risk of social isolation or socially isolated defined by the following:

**Table 2:** Selection criteria

Inclusion criteria
- Patient aged 70 years or older - Socially isolated or at risk of social isolation (m-MOS-SS under 80% [13] and/or isolated patient (caregiver living more than 50 km from the patient) and/or primary caregiver to his/her spouse [the spouse who has a limited autonomy (neurodegenerative disease, cancer, etc.] implying regular medical care for at least 3 months) - G8 score ≤14 - Performance status ≤2 - Autonomous (ADL score ≥4) - Life span >6 months - Metastatic or locally advanced tumor histologically proven from: colon, rectum, pancreas, breast, ovary, lung, superior aerodigestive path, prostate, urothelium - Being treated for first line of chemotherapy or radio-chemotherapy - Patient affiliated to the French care system protection - Informed consent given
Exclusion criteria
- Patient aged under 70 years old - Patient who has to be hospitalized straight away - Patient with psychiatric troubles or advanced dementia - Protected adults under guardianship or curatorship - Patients with a second cancer except for cervical cancer - Patients unable to complete the QoL questionnaire without help - Patient to be directed immediately into a rehabilitation and recuperative care service to benefit from treatment.

- An m-MOS-SS score under 80%: auto-questionnaire composed of 8 items derived for the Medical Outcomes Study Social Support Survey (MOS-SS) and validated for elderly patients treated for cancer pathologies [13]
- And/or isolated patient, living alone with a primary caregiver (designated by the patient), living more than 50 km from the patient’s home
- And/or patient him(her)self being primary caregiver to a spouse whose autonomy is limited (neurodegenerative disease, cancer, etc.), implying regular medical care for at least 3 months

The main exclusion criteria are patients under 70 years old, with a WHO score equal or superior to 3, patients unable to understand/fill out self-administered questionnaires, patients with a concomitant second oncologic pathology or in clinical relapse of a second cancer, except for cervical cancer or ancient oncologic pathologies (more than 5 years ago), and patients to be directed immediately into a rehabilitation and recuperative care service to benefit from treatment.

### Groups

#### Control group: usual care management

The patients in the control group will follow the standard care pathway: after the diagnosis is announced, a personalized treatment plan will be built. As recommended by the SIOG and INCa [8], each patient more than 70 years old will be screened for geriatric frailties by the G8 scale. Patients with a score ≤14 will be addressed to a geriatrician in order to benefit from a full CGA [9]. Patients referred by the geriatrician, or the oncologist on the recommendation of the geriatrician, who are detected as at risk of isolation or isolated will be addressed to a social worker as planned by the standard care pathway. The patients will receive the usual oncologic care management consisting of a monthly visit with the oncologist, who will check clinical and biological toxicity. At 3 and 6 months, along with clinical and biological toxicity, the oncologists will evaluate dose response-intensity, tumor response, functional status and autonomy (self-administered questionnaires dedicated to the study), nutritional status, and QoL.

#### Experimental group

The patients in this group will follow the same standard care management, up to the visit with the social worker. At this point the social worker is in charge of establishing a Program of Social intervention associated with techniques of Domotic and Remote assistance (PS-DR): besides the usual social management, the social worker will perform a monthly telephone follow-up of the patient, verify that all the geriatrician recommendations are instituted (nurse, physiotherapist visits, etc.), and coordinate the setup of the domotic care package. Above all, the social workers will ensure the maintenance of the autonomy of the patients and allow them to be actors in their life projects. To avoid missing follow-up, the social worker will be provided with the contact information of every supporting staff member caring for the patient (nurse, home help, general practitioner, etc.). Furthermore, the homes of patients benefiting from social follow-up are connected with remote assistance 24/7; the device is not only used for the patient to call for help but can be remotely activated to contact the patient in case of prolonged inactivity of the sensors. Finally, the protocol states the possibility for the social worker to make home visits if necessary. The domotic care package is composed of a remote assistance transmitter (7 days/week and 24 hours/day available contact), an automated portable fall detection device, smoke and carbon monoxide detectors, and activity monitors associated to an autonomous automated lighting system. This system will provide round-the-clock help to the patient in association with usual care dispositions (nurse, physiotherapy, housekeeping help). The intervention will last 6 months following the initial visit.

### Recruitment and follow-up

#### Screening, inclusion, and randomization (*t*_*–1*_)

The eligible patients will be identified by the investigators of each participant center. Patients more than 70 years old screened with a G8 score ≤14 will be addressed to a geriatrician and benefit from a full CGA. The geriatrician will screen at-risk or isolated patients (as described above). Patients who meet all the inclusion criteria will be included. After completing the consent form, they will be randomized into one of the two groups as follows: computer-generated randomized lists will be drawn up before the beginning of the study, using a permuted block design, under the responsibility of the clinical research unit (AP-HM). The randomization will be stratified by center (1:1 allocation ratio).

#### Follow-up and data collection (T0 to T7)

The evaluation will be performed at six different time points: first treatment visit (T0, beginning of the intervention) and intermediary visits (T1, T2, T3, T4, and T5, one month after the previous visit +/– one week, according to oncological treatment). The end of treatment (T6) will take place 6 months after T0, and a T7 will take place 12 months after T0 for survival follow-up.

The total duration of the study will be 42 months, the recruitment period will be 30 months, and the patient follow-up period will be 12 months.

The study procedure and data collection are detailed in Table 3.

**Table 3:** Study procedure

	Inclusion		Intervention implementation		6 months intervention												Close-out	
	t_-1_		T0		T1		T2		T3		T4		T5		T6		T7	
Exposed (E) or Non-exposed (NE) patients	E	NE	E	NE	E	NE	E	NE	E	NE	E	NE	E	NE	E	NE	E	NE
Enrollment:																		
Initial oncologist consult (geriatric frailties screening G8 scale)	X	X																
Comprehensive Geriatric Assessment (CGA) (social isolation evaluation, eligibility screening)	X	X																
Informed consent	X	X																
Allocation	X	X																
Social worker initial evaluation (SE)			SE	SE														
Domotic pack installation			X	X														
Intervention:																		
Oncologist consult and treatment			X	X	X	X	X	X	X	X	X	X	X	X	X	X	X	X
Social worker telephone follow-up (TF)					TF		TF		TF		TF		TF		TF			
Domotic and remote assistance care package					X		X		X		X		X		X			
Assessments:																		
Autonomy (ADL + IADL); Nutritional and functional status evaluation	X	X							X	X					X	X		
Tumor response (RECIST 1.1)									X	X					X	X	X	X
Dose response-intensity (Bonadonna criteria)									X	X					X	X		
Treatment toxicity (NCI-CTCAE)					X	X	X	X	X	X	X	X	X	X	X	X		
QoL evaluation (EORTC QLQ-C30 + ELD 14 module)			X	X					X	X					X	X		
Survival																	X	X

*t*_*-1*_ inclusion; *T0* intervention implementation (15 days or less after t_–1_, *T1* 1 month after T0 +/– 1 week, *T2* 1 month after T1 +/– 1 week, *T3* 1 month after T2 +/– 1 week, *T4* 1 month after T3 +/– 1 week, *T5* 1 month after T4 +/– 1 week, *T6* 1 month after T5 +/– 1 week, *T7* 12 months after T1)

### Endpoints/evaluation criteria

#### Primary endpoint

The primary endpoint is the QoL assessed at T3 according to the first line of treatment (usually 3 months after T0) using the European Organization for Research and Treatment of Cancer (EORTC) QLQ-C30 questionnaire. This is a 30-item questionnaire composed of five functional scales (physical, role, emotional, cognitive, and social), nine symptom scales and single-symptom items, plus a global health status scale [16]. The scores on each scale/item range from 0 to 100. A high score on the functional scale represents a high/healthy level of functioning, a high score on the global health status scale represents a high QoL, and a high score on the symptom scale represents a high level of symptomatology. The global health score will be the primary endpoint.

#### Secondary endpoints

QoL assessment will be complemented by the following endpoints:

- EORTC QLQ-C30 at 6 and 12 months post-randomization.
- The EORTC QLQ-ELD14, an additional module for measuring QoL in patients aged ≥70 years in oncology studies, was added to the core questionnaire and comprises 14 questions assessing elderly-specific needs analyzed on five scales (mobility, worries about others, future worries, maintaining purpose, and illness burden) and two single items (joint stiffness and family support) [17]. High scores indicate a high level of problems.
- Time to treatment failure will be assessed as the delay between inclusion and treatment failure whatever may the cause be. Treatment failure will be assessed as defined by the Response Evaluation Criteria In Solid Tumors (RECIST) 1.1 criteria [18].
- Toxicity at the end of the treatment will be evaluated using the NCI-CTCAE (National Cancer Institute – Common Terminology Criteria For Adverse events, version 4.0)
- Compliance will be evaluated by observance of the oncologic treatment defined by the following parameters: the treatment completion in terms of number of chemotherapy cycles received out the number intended, will be compared between the two groups; the dose response-intensity (DRI) defined by the relative amount of treatment delivered by time units in relation to a standard treatment chosen arbitrarily or in relation to a combined treatment. A DRI <85% indicates a significant diminution according to the Bonadonna criteria [19].
- Autonomy will be assessed by the Katz and Lawton scales respectively measuring the patient’s ability to perform activities of daily living (ADL Scale) and Instrumental Activities of Daily Living (IADL Scale) at 3 and 6 months. Patients unable to perform at least one activity will be considered as dependent either in daily living activities or instrumental activities, except for urinary incontinence. Patients enrolled in the study must present an ADL score higher or equal to 4 (out of 6). Decline in autonomy will be assessed as the number of activities at 3 and 6 months compared to the baseline.
- Overall survival will be calculated as a function of time between inclusion and authenticated death of the patient (death certificate or hospital reporting) at 6 and 12 months.
- Progression-free survival will be calculated as a function of time between inclusion and authenticated cancer progression according to the RECIST 1.1 criteria [18] at 6 and 12 months.
- Social isolation will be measured by the m-MOS-SS (modified Medical Outcomes Study Social Support Survey) scale and the following items: housing, living place (urban, suburban, or rural), marital status, lifestyle (single or family with children or spouse), resource person and caregiver geographical distance with children or the primary caregiver, medical and social, and recreational pursuits, desire for life, and motivation for treatment.
- The number of unplanned re-hospitalizations will also be documented.

### Statistical considerations

#### Sample size, power, and statistical methods

The sample size was determined to obtain a 90% power to detect a clinically significant 10-point difference (standard deviation 25) in QoL at 3 months between the two groups. This difference was based on norms provided by the EORTC (http://groups.eortc.be/qol/manuals) with the threshold for statistical significance set at a *p* value of 0.05. Because of the severity of these patients’ pathology and the high risk of mortality of metastatic cancer combined with an aging population [6] and social isolation [20–24, 25], we assumed that a potential 20% of patients will be lost to follow-up. These calculations showed that 320 patients are needed (160 per group).

#### Data analysis

The data will be analyzed using SPSS version 17.0 software. The patients found to be eligible but not included in the study will be described and compared with the included patients. The patients who present at least one of the following conditions will not be included in the final analysis: patients inappropriately included despite providing consent and patients who remove their consent. The full analysis population (including all subjects who will be randomized and at least evaluated at T1) will be used in the primary analysis, and the per protocol population (including all subjects who will be randomized and will not have major protocol deviations) will be used in the secondary analysis to assess the robustness of the results. No interim analysis is planned. The normality of these parameters will be estimated using frequency histograms and the Shapiro test. The baseline parameters will be presented per group (control and experimental).

The QoL scores will be calculated according to the developers’ consideration. The general health score at T3 will be compared between the two groups using Student’s *t* test for continuous variables. Other QoL scores will be similarly compared. Changes between each initial score (T0) and the score at T3 and at T6 will be compared between the two groups, and the analysis of variance for repeated measurements will be performed to compare the changes in the scores over time between the two groups. Multivariate analysis using linear regression models will be performed to determine variables potentially linked to QoL. Variables relevant to the models will be selected based on their clinical significance (including group). The final models will estimate the beta standardized coefficients. The secondary endpoints will be compared between the two groups (chi-squared test or Fisher’s exact test for categorical variables and Student’s *t* test for continuous variables). All of the tests will be two-tailed with a 5% significance level.

## Discussion

To date, no randomized study has determined the impact of a social intervention associated with domotic and remote assistance techniques on the QoL of elderly patients being treated for locally advanced or metastatic cancer. We wish to demonstrate that strengthening social support is key to maintain the patient’s autonomy and limit unplanned hospitalization while improving the isolated elderly patient’s QoL. Domotic and remote assistance techniques can be a part of the response to social isolation of the elderly. They already demonstrated their benefit according to the Esoppe study: the risk of fall was divided by 3 and autonomy was improved [14]. Effects of telecare on unplanned hospitalizations are contradictory [26–28], but the Telecare Demonstrator study [15, 26], while demonstrating no significant effects on re-admissions, shows a relative improvement in QoL.

These techniques are clearly helpful but cannot replace human contact with resource persons at home [29, 30]. We think that integrated autonomous systems must be a part of a larger multidisciplinary disposition of home care and that it would especially benefit isolated patients. After discussion with the members of the steering committee, we decided to have two interventions in the experimental group (PS-DR: home automation and remote assistance associated with a monthly telephone follow-up); as part of the usual care and social follow-up, we concluded that a social worker is the best choice to coordinate the various teams around the patient and manage the monthly patient follow-up.

Considering the current recommendations of the SIOG and the INCa to have a geriatric assessment with a G8 score ≤14, it was also essential that the two groups benefit from initial geriatric opinions and recommendations. Moreover, the geriatric assessment will allow us to better evaluate the inclusion criteria (social isolation, autonomy) as well as baseline primary and secondary endpoints (QoL, nutritional and functional status of the patient). To demonstrate that the PS-DR is efficient in maintaining autonomy and preventing re-hospitalization, we had to consider a specific profile of patients. Patient who would not be able to handle the oncologic treatment or fill out the self-administered forms (i.e., QoL questionnaires) could not be enrolled in the study. For these reasons we chose to include patients who were in good general heath (WHO score ≤2) and autonomous (ADL ≥4) and to exclude patients with psychiatric troubles or advanced dementia.

Quality of life is a subject of increasing interest on the part of oncologists: it is increasingly used as the endpoint of efficacy and tolerability of treatment, or as a therapeutic decision tool [31–33]. As defined by the WHO, it is “the perception of an individual's place in existence, in the context of the culture and value system in which he lives, in connection with its objectives, expectations, standards and concerns. This is a broad conceptual field, covering in a complex way the person's physical health, psychological state, level of independence, social relationships, personal beliefs and relationship to the characteristics of its environment.” In elderly patients with cancer, QoL meets the same definitions as in the young: it is an indicator of health and is particularly interesting and potentially more relevant than the sole assessment of survival. Indeed, its analysis allows a broader assessment of disease and treatment impact on the physical, mental, and social health of the elderly patient, considering his environment, his expectations, and his experience [34–36]. These considerations motivated our choice of primary endpoint: QoL at 3 months, as it also allows us to take into account the limited life expectancy of patients with locally advanced or metastatic tumors, including lung and pancreatic cancer. Several factors have an impact on QoL, including functional status, comorbidities, and depression [33]; as such, QoL can be measured by various consensual questionnaires such as the SF-36 questionnaire or the EORTC QLQ-C30 scale. We settled on the EORTC QLQ-C30 scale modulated by EORTC QLQ-14 ELD for its specificity to our population (elderly cancer patients [17]).

Social support has often been assessed in terms of survival or progression [5, 37] or in QoL of survivor patients [3, 38]. Very little is known about the relation between social support and QoL during the oncologic treatment, and there is no interventional study that is comparative and randomized on the influence of the strengthening of social support in socially vulnerable elderly persons being treated for cancer.

We chose to randomize patients into two groups to have a reliable methodology and a methodologically valid conclusion. In light of the current literature in the field, the experimental design chosen corresponds to bringing the highest level of evidence. The choice of open mode is the only possible one within the framework of this project, because it is impossible for the subject and/or the physician investigator not to know the assigned group. However, in order to minimize bias related to “open” mode, the statistical analysis will be performed blind.

Although the PS-DR setup is less expensive than unplanned hospitalization or admission in a support care facility, installation of a home automation system for the 160 patients of the exposed arm has a cost. To meet both clinical and financial requirements, it has been decided that the intervention would last 6 months (end of the first line of treatment for most locally advanced or metastatic cancers), at the end of which each domotic care package will be uninstalled. The steering committee discussed the ethics of removing the pack, in particular the possibility, for patients who ask for it, to maintain the system as long as they lived. Two major arguments prevented us from allowing such dispositions. Firstly, for methodological reasons, including the point of survival at 12 months, it is important that the living conditions of patients in each arm be as reproducible as possible. Secondly, loss of chance in the control group is not proven—the study would be unethical if that were the case—and all the usual care support (nurse, physiotherapy, housekeeping) also present in the control group will remain in place. We therefore concluded it was important to withdraw the packs in all patients beyond 6 months, in order to preserve the homogeneity of the experimental group.

If our hypothesis is verified, we could offer a new support scheme for these susceptible patients and allow them to receive more intensive treatment with better adherence and less toxicity.

In addition, cancer management is a time of many changes for the patient, and there is little data available for the elderly to better understand why, for equivalent severity, some patients’ QoL degrades rapidly when it is maintained among others [39–43].

In conclusion, the results of this randomized trial are expected to confirm that a PS-DR may be an interesting care management strategy in isolated elderly patients with cancer.

### Trial status

At the time of manuscript submission, the status of the trial is ”not yet recruiting.”

## Additional file

Additional file 1: SPIRIT checklist items. (DOC 255 kb)

## Abbreviations

ADL: Activities of daily living
ANSM: Agence Nationale de Sécurité du Médicament
AP-HM: Assistance Publique-Hôpitaux de Marseille
CONSORT: Consolidated Standards of Reporting Trials
DRI: Dose response-intensity
EORTC QLQ-14 ELD: European Organization for Research and Treatment of Cancer complementary module of quality of life form C30, ELD-14 dedicated to elderly cancer patients
EORTC QLQ-C30: European Organization for Research and Treatment of Cancer quality of life form C30
G8 ONCODAGE: scale for detection of geriatric frailties in cancer patients over 70 years old
m-MOS-SS: modified Medical Outcomes Study Social Support Survey
PS-DR: Program of Social intervention associated to Domotic and Remote assistance techniques
QoL: Quality of life
RCT: Randomized controlled trial
WHO: World Health Organization
